# Primary anorectal melanotic melanoma: a case report

**DOI:** 10.1186/s13256-026-06224-3

**Published:** 2026-06-17

**Authors:** Kaitlyn Brown, Sofia Sirocchi, William Frank Willett

**Affiliations:** 1 Philadelphia College of Osteopathic Medicine - Georgia Campus, 625 Old Peachtree Rd NW, Suwanee, GA 30024 USA; 2 Philadelphia College of Osteopathic Medicine - South Georgia Campus, 2050 Tallokas Rd, Moultrie, GA 31768 USA; 3 St. Francis - Emory Healthcare, 2122 Manchester Expy, Columbus, GA 31904 USA

**Keywords:** Anorectal melanoma, Anorectal malignancy, Primary mucosal melanoma, Case report

## Abstract

**Background:**

Anorectal melanoma (AM) is a rare and aggressive malignancy that is often advanced at the time of diagnosis. This case demonstrates the importance of a prompt colonoscopic evaluation, especially in older patients presenting with new gastrointestinal symptoms, and underscores the need for further research establishing a standardized treatment protocol.

**Case presentation:**

We report a case of a 71-year-old white male who was referred to gastroenterology after noticing increased frequency of bright red blood per rectum following bowel movements. A subsequent colonoscopy revealed a 1.5-cm darkly pigmented, irregular mass in the anorectal region. The lesion was excised and sent for microscopic evaluation which disclosed highly malignant spindle and epithelioid cells producing a large amount of brown pigment. A diagnosis of anorectal melanoma was confirmed with positive immunohistochemical staining for S-100, HMB45, and Melan-A. A computed tomography (CT) scan of the abdomen and pelvis showed no evidence of metastasis. The patient then underwent a transanal resection of the area where the tumor was found. No residual melanoma was seen in the resected specimen. Follow-up evaluations over about 2 years have shown no evidence of recurrence.

**Conclusions:**

The positive outcome of this case highlights the importance of timely recognition as well as the ongoing need to explore and refine effective treatment options for AM.

## Background

Anorectal melanoma (AM) is a rare neoplasm, accounting for less than 1% of all colorectal malignancies [1]. AM is twice as likely to occur in women than in men and is typically seen in white patients with a mean age of 63 [1]. Patients may present with nonspecific complaints, such as rectal bleeding (most common), anal pain, or changes in bowel habits [1, 2]. Diagnosis is often delayed, as initial symptoms are similar to those of hemorrhoids or other benign conditions [3].

The appearance of AM is usually polypoid, with only 20% of lesions showing obvious melanin pigmentation [3]. Microscopic appearance is similar to cutaneous melanoma, featuring nests of atypical melanocytes and frequent mitoses. Deposits of brown–black melanin may also be present as fine pigment in the tumor cells or as clumps in melanophages [1]. Immunohistochemical staining typically results in S-100, Melan-A, and HMB45 positivity, with Melan-A being most sensitive and specific for diagnosis [1].

The prognosis of AM is poor, with many patients having distal metastasis at the time of diagnosis [4]. Due to the rarity of this neoplasm, standardized treatment protocols are lacking [5]. AM responds poorly to chemotherapy and radiation; therefore, surgical treatment is necessary with either wide local excision or abdominoperineal resection (APR) [4]. However, there is debate as to which approach is best, and since most patients with this disease will have a limited life expectancy, quality of life after surgery is an important consideration [5, 6]. This case report describes a case of anorectal melanoma with a positive outcome, highlighting the importance of a timely diagnosis and the ongoing need to establish a standardized treatment protocol.

## Case presentation

**Fig. 1:**
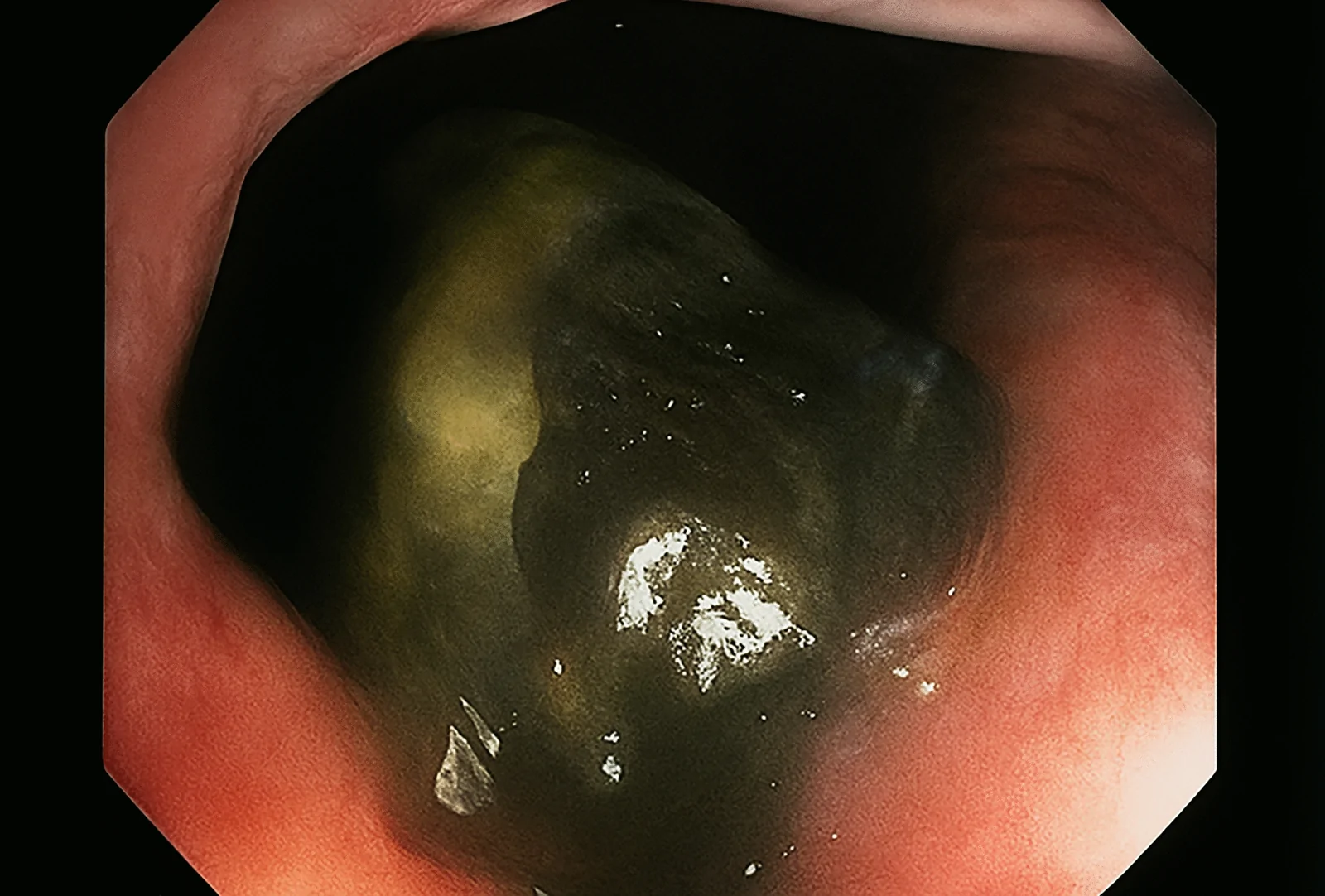
Colonoscopic image showing a black polypoid mass in the anorectal region measuring 1.5 cm

The patient is a 71-year-old white male with a past medical history of colon polyps, non-alcoholic fatty liver disease, atypical atrial fibrillation, hyperlipidemia, benign essential hypertension, and type 2 diabetes mellitus who was referred to gastroenterology for increased frequency of bright red blood per rectum noticed on tissue paper following bowel movements. Physical examination, which included cardiovascular, pulmonary, abdominal, and extremity evaluation, was unremarkable at this time. Rectal examination revealed no evidence of external hemorrhoids, perianal fissures, or perianal fistulas as well as good rectal tone with no palpable rectal masses. A subsequent colonoscopy revealed a 1.5-cm irregular, black polypoid mass in the anorectal region (Fig. 1). The lesion was excised and sent for microscopic analysis.

**Fig. 2:**
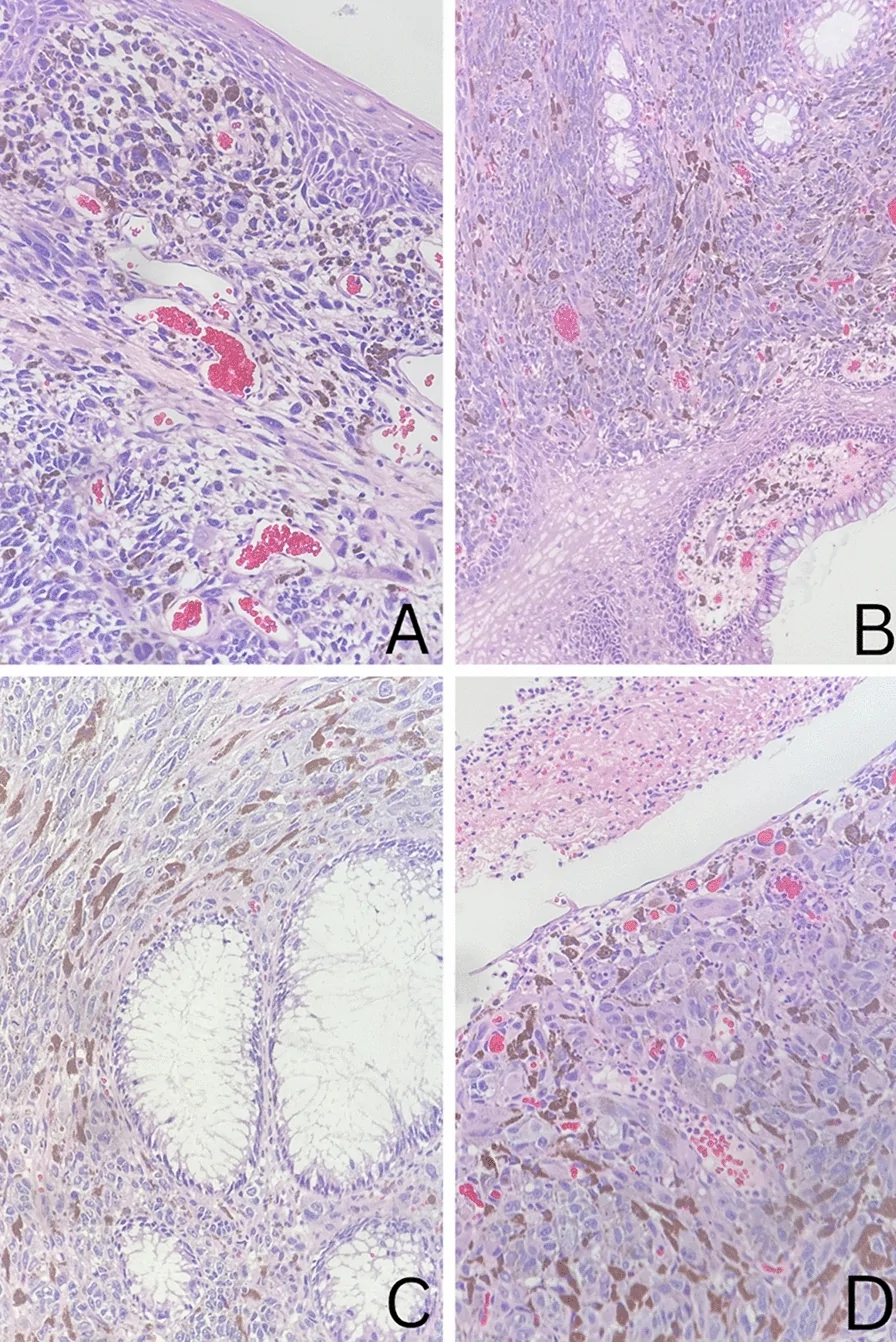
Hematoxylin and eosin (H&E) stained sections of excised anorectal mass **A** Highly atypical cells with pleomorphic nuclei, large nucleoli, and numerous mitotic figures underneath the squamous mucosa of the anus with prominent melanin pigment present. **B** Same cells with high-grade nuclear atypia, mitoses, and spindle morphology at the squamocolumnar junction of the dentate line. **C** Pigmented melanoma cells invading the rectal mucosa. **D** Melanoma cells associated with a fibrinopurulent membrane indicating adjacent ulceration

Microscopic examination revealed attenuated and eroded squamous epithelium under which a large mass of infiltrative, highly malignant spindle and epithelioid cells producing a large amount of brown pigment was observed (Fig. 2). The cells showed large, irregular nuclei with scattered large nucleoli and mitoses. Immunohistochemical staining with S-100, Melan-A, HMB45, and Ki-67 were performed, revealing positive S-100, Melan-A, and HMB45 and a Ki-67 of 10% nuclear positivity. Based on clinical information, microscopic findings, and staining results, the diagnosis was determined to be a primary mucosal melanotic melanoma involving anal and rectal mucosa. The possibility of metastatic cutaneous melanoma (CM) was ruled out.

A computed tomography (CT) scan of the abdomen and pelvis showed no metastatic disease, indicating stage I (localized) disease. The patient then underwent a transanal resection of the area where the melanoma was found. No residual melanoma was seen in the resected specimen, implying that the tumor was completely excised endoscopically. The patient has had close surveillance with sigmoidoscopies and CT scans since and there has been no evidence of recurrence over the course of approximately 2 years.

## Discussion

The exact etiology of AM is currently unknown. All melanomas arise from the melanocyte and in the rectum, the anal transition zone and squamous zone are normal areas for melanocytes to exist [7]. Because melanocytes are derived from the neural crest or from the mucocutaneous junctions to the cutis, it has been theorized that AM may arise from Schwannian neuroblastic cells of the autonomic intestinal innervation system or from cells that conduct the amine-precursor uptake and decarboxylation system in the gut, as these cells are from the neuroectoderm [8]. Because melanocytes have significant antioxidant and immunogenic properties, it has also been hypothesized that AM favors an environment of oxidative stress and/or compromised immune system [8, 9]. In fact, there is indirect evidence that infection with human immunodeficiency virus (HIV) may be implicated as a risk factor [10]. There also seems to be a higher incidence of AM in patients with human papilloma virus (HPV) [7]. In addition, AM is more common in women. The suspected reason for this is hormonal in nature, as estrogens increase the number of melanocytes and can alter melanin content [8]. Of note, unlike CM, sun exposure is not a risk factor for mucosal melanoma and individuals with darker skin actually have a higher incidence of AM [9].

Compared to CM, primary mucosal melanoma (PMM), of which AM is a subtype, has several unique genetic features [8]. There is a lower BRAF mutation rate in PMM, which indicates that BRAF inhibitors may not be helpful in treatment [8, 11]. There is also a lower NRAS, GNAQ, and GNAII mutation rate in PMM compared to CM [8]. However, it has been reported that AM shows a higher rate of KIT mutations, with one study reporting > 30% of cases [12]. c-KIT stimulates growth, differentiation, migration, and proliferation of melanocytes and activates other signaling cascades that cause cancer spread, such as phosphoinositide 3-kinase (PI3K)/AKT, mitogen-activated protein kinase (MAPK), and Janus kinase (JAK) [8]. Some trials have shown clinical benefit with imatinib, a tyrosine kinase inhibitor, in patients with metastatic PMM with activating KIT mutations; however, secondary resistance eventually develops [13]. Additional mutations identified include recurrent mutations in the tumor suppressor NF1 in about 20% of cases as well as SF3B1 mutations [13].

Due to nonspecific symptomatology, diagnosis of AM is often delayed. Anal pain, rectal bleeding, and changes in bowel movements are all symptoms that can easily be mistaken for hemorrhoids or another benign condition [3]. As with any malignancy, early detection of AM is key to improving the chance of survival. Since there are no clinical features unique to AM, a thorough evaluation must be performed, especially in older patients, which should include a rectal examination, endoscopic ultrasound, and proctosigmoidoscopy [7, 8]. Another complicating factor in the diagnosis of AM is its potential to appear nonpigmented, which on initial impression may be mistaken for a benign polyp [3]. This makes microscopic evaluation essential. AM has four histologic subtypes, including epithelioid, spindle-cell, lymphoma-like, and pleomorphic [14]. However, depending on the subtype, AM can still be confused with another type of malignancy. For example, spindle-cell AM resembles and can be mistaken for a rectal gastrointestinal stromal tumor [14]. Therefore, immunohistochemical staining is necessary for making the final diagnosis, with Melan-A being the most sensitive and specific stain [1]. To assess for loco-regional and systemic involvement, 18F-fluorodeoxyglucose positron emission tomography (18F-FDG–PET)/CT and CT with contrast can be used, with the former being the best option for staging the cancer, which helps formulate a treatment plan [8].

Because of the rarity of AM, a standardized treatment protocol has not been determined [5]. Since response to chemotherapy and radiation is poor, surgery is currently the cornerstone of treatment. The two main treatment options include wide local excision of the primary tumor with or without radiotherapy, or APR with or without inguinal and pelvic lymphadenectomy [6, 15]. While there is conflicting information in the literature regarding which treatment is more effective, the goals remain the same: locally controlling the tumor and preventing recurrence [6]. In general, wide local excision seems to be the treatment of choice for small tumors, while APR is preferred for large, obstructing lesions [15]. Suggestions for future research on the treatment of AM include investigating new drug regimens or treatment modalities, such as immunotherapy. These include immune checkpoint inhibitors and tyrosine kinase inhibitors, as well as vaccines that induce humoral immunity and enhance cellular immunity, inflammatory cytokines, and immune modulators [8, 9].

Regardless of treatment, prognosis remains poor, with 5-year survival rates of 6–22% [1]. Delayed diagnosis combined with rapid progression means that patients may have rectal wall infiltration, lymph node involvement, and/or distant metastasis at the time of diagnosis, all of which contribute to an unfavorable prognosis [3]. One study revealed that out of all types of mucosal melanomas, anorectal melanoma carried the highest probability of metastasizing to the brain, further supporting the aggressive nature of this disease [16]. Histologically, tumor thickness, tumor necrosis, and perineural invasion are also associated with a poorer prognosis [7]. Anal-only involvement has been shown to be associated with increased rates of recurrence when contrasted with rectal-only involvement and anorectal involvement; however, recurrence remains a concern regardless of tumor location [8]. Histologic type of the tumor has also shown an association with recurrence with pure epithelioid histology being more likely to recur when compared to the other types [7].

## Conclusions

AM is a rare and aggressive disease. Although the gastrointestinal tract is an uncommon site for melanoma, clinicians must still maintain an index of suspicion. Nonspecific clinical presentation means that diagnosis is often delayed and disease is advanced at the time of detection. The positive outcome of this case demonstrates the importance of a prompt colonoscopic evaluation, especially in older patients with new gastrointestinal complaints, as outcomes correlate with timely recognition. Given the rarity of this neoplasm, no formal treatment protocol exists. While surgery remains the primary treatment option, this case report underscores the need for further research into a standardized treatment protocol and the role of adjunctive chemotherapy and immunotherapy. These emerging modalities show promise in improving outcomes and are likely to be refined as our understanding of this malignancy advances.

## Data Availability

Not applicable as no datasets were generated or analyzed.

## Abbreviations

AM: Anorectal melanoma
APR: Abdominoperineal resection
CM: Cutaneous melanoma
H&E: Hematoxylin and eosin
CT: Computed tomography
PMM: Primary mucosal melanoma
PI3K: Phosphoinositide 3-kinase
MAPK: Mitogen-activated protein kinase
JAK: Janus kinase
18F-FDG–PET: 18F-fluorodeoxyglucose–positron emission tomography
